# Bem Comer and indigenous pathways of healing: prevalence of overweight and food coloniality among Xukuru do Ororubá Brazil

**DOI:** 10.3389/fpubh.2026.1788986

**Published:** 2026-03-16

**Authors:** Heitor Victor Santos da Silva, Renata Gomes de Lima Melo, Laryssa Rebeca de Souza Melo, Pedro Israel Cabral de Lira, Israel Cavalcante Soares, Juliana Souza Oliveira

**Affiliations:** 1 Department of Nutrition, Center for Health Sciences, Federal University of Pernambuco, Recife, Pernambuco, Brazil; 2 Federal Institute of Amazonas, Tefé, Amazonas, Brazil; 3 Pernambuco Faculty of Health, Recife, Pernambuco, Brazil; 4 Vitória Academic Center, Federal University of Pernambuco, Vitória de Santo Antão, Pernambuco, Brazil

**Keywords:** food coloniality, food sovereignty, indigenous adolescents, indigenous healing, overweight, territory

## Abstract

**Background:**

Indigenous adolescents are increasingly exposed to nutritional transitions resulting from historical processes of food coloniality, territorial disruption, and transformations in traditional food systems. Territory plays a central role in Indigenous health, shaping food practices, social organization, and systems of care. However, evidence on overweight among Indigenous adolescents remains scarce, particularly analyses that integrate epidemiological findings with Indigenous epistemologies of healing and well-being.

**Objective:**

To estimate the prevalence of overweight among adolescents of the Xukuru do Ororubá people and to analyze its association with socioeconomic conditions, lifestyle factors, and body weight perception, interpreting the findings within the territorial and cultural context in which Indigenous systems of care are produced.

**Methods:**

Cross-sectional analytical study grounded in a critical public health framework, in which quantitative epidemiology is used as an interpretative tool rather than an epistemologically neutral endpoint. The study was conducted in 2022 with self-identified Indigenous adolescents aged 12 to 18 years residing in villages within the Xukuru do Ororubá territory, Pernambuco, Brazil. Data were collected through structured questionnaires and anthropometric measurements. Overweight was defined using BMI-for-age z-scores according to WHO criteria. Associations were estimated using Poisson regression with robust variance. The study followed ethical principles for research with Indigenous peoples, including community authorization and adherence to CARE principles.

**Results:**

A total of 225 adolescents participated. The prevalence of overweight was 14.2%, higher among females. In the multivariable analysis, overweight was associated with household sanitation conditions, maternal schooling, school shift, and dissatisfaction with body weight. Lifestyle variables were not independently associated.

**Conclusion:**

Overweight among Xukuru do Ororubá adolescents reflects na interplay between socioeconomic vulnerability, school organization, body perception, and structural transformations affecting Indigenous territories and food systems. By articulating epidemiological evidence with Indigenous knowledge of Bem Comer and territorial healing practices, this study contributes to a decolonial interpretation of adolescent health in Indigenous contexts.

## 1 Introduction

Indigenous peoples have historically engaged in intense processes of political mobilization and collective organization to defend their rights and articulate their demands. Unlike other social movements, Indigenous struggles for land are grounded in a broader understanding of territory, conceived not merely as a physical or legal space, but as a living environment where the material, cultural, spiritual, and political dimensions of life are reproduced. Territory, therefore, constitutes a fundamental determinant of health, care, and food systems among Indigenous peoples (1).

Within Indigenous worldviews, the human body is inseparable from territory, collectivity, and the spiritual world. Eating, cultivating the land, and caring for the body are understood as interconnected practices within a single life system, in which health, spirituality, and nature are deeply intertwined. Food, in this context, is not limited to nutritional intake, but embodies relationships of belonging, reciprocity, and balance among all beings that inhabit the territory. Body, food, and land thus form interdependent dimensions of Indigenous systems of care and well-being (2, 3).

However, this ancestral relationship between body, territory, and food has been historically disrupted by colonial and neocolonial processes that profoundly reshaped Indigenous ways of living. The replacement of autonomous food systems with exogenous models, characterized by the introduction of ultra-processed foods, the erosion of traditional knowledge, and the weakening of food sovereignty, has generated ruptures that are reflected both in cultural practices and in contemporary health profiles (4). From this perspective, overweight among Indigenous peoples cannot be understood merely as an isolated nutritional outcome, but rather as an expression of food coloniality that operates on bodies, territories, and identities.

Under these conditions, the Indigenous body itself becomes a territory marked by historical, political, and symbolic disputes. Coloniality extends beyond land dispossession, exerting control over eating practices, social rhythms, body perceptions, and modes of care. As a result, overweight emerges as a complex phenomenon associated with the dismantling of traditional food systems, the forced reorganization of daily life, and the imposition of Western dietary and aesthetic norms that undermine Indigenous conceptions of the body and of well-being (5).

According to the 2022 Brazilian Demographic Census, the Indigenous population in Brazil comprises 1,693,535 individuals, representing 0.83% of the total population, with the majority residing in the North and Northeast regions (6). In the state of Pernambuco, the Xukuru do Ororubá people constitute one of the largest Indigenous populations, with approximately 8,000 individuals living in 31 villages distributed across the Serra, Agreste, and Ribeira regions. The Xukuru territory was officially recognized in 2001 after a prolonged struggle for land restitution and remains a central axis of identity, spirituality, and care practices (7, 8).

The history of the Xukuru do Ororubá people is marked by resistance to colonial processes initiated between the late eighteenth and early nineteenth centuries, whose effects remain present in collective memory and contemporary practices of (re)existence. Internationally recognized as the “Tree-Bird People,” the Xukuru maintain a profound relationship with traditional agriculture and the natural environment, promoting the so-called “Sacred Agriculture” and the practice of *Bem Comer* (“Eating Well”) as foundations of a way of life that integrates food, spirituality, territory, and healing (9). These practices function not only as subsistence strategies, but also as Indigenous systems of care and health promotion.

Despite the resilience of these traditional systems, the impacts of globalization, market expansion, and more recently the COVID-19 pandemic have intensified changes in food consumption patterns and lifestyles, particularly affecting Indigenous adolescents. This life stage is marked by heightened exposure to external influences and social transformations, placing Indigenous youth at the intersection of ancestral knowledge and contemporary pressures, and generating specific vulnerabilities related to nutrition and health (10–12).

Overweight is currently one of the most pressing public health challenges worldwide, with increasing prevalence across age groups. Among Indigenous peoples, its emergence is closely linked to structural processes such as territorial loss, weakened food sovereignty, and disruptions in traditional ways of living. In adolescents, overweight may also lead to early cardiometabolic alterations, including hypertension, dyslipidemia, and type 2 diabetes, increasing health risks throughout the life course (13–15).

Within this context, evidence on overweight among Indigenous adolescents in Brazil remains scarce, especially studies that articulate epidemiological findings with Indigenous systems of healing and well-being. Most investigations focus on children or adults, leaving significant gaps in understanding how nutritional and bodily transformations unfold during Indigenous adolescence (16–24). Existing studies among Kamayurá and Kaingáng adolescents suggest that overweight is an emerging concern, reflecting broader changes in dietary patterns and physical activity within Indigenous communities (25–27).

In this context, evidence on overweight among Indigenous adolescents in Brazil remains limited, particularly studies that interpret epidemiological findings through Indigenous knowledge systems of care and healing. Most existing research focuses on children or adults, leaving important gaps in understanding how nutritional and bodily transformations unfold during Indigenous adolescence.

Therefore, this study aimed not only to estimate the prevalence of overweight among Xukuru do Ororubá adolescents but also to interpret these findings through Indigenous conceptions of Bem Comer, territoriality, and healing. By positioning epidemiology as a starting point for dialogue rather than as an epistemologically neutral framework, this study seeks to bridge quantitative public health analysis with Indigenous epistemologies of care.

## 2 Methods

### 2.1 Study design and setting

This school-based, cross-sectional analytical epidemiological study was conducted using conventional quantitative epidemiological procedures. The quantitative findings were subsequently interpreted within a critical public health framework, informed by structural and decolonial perspectives, to contextualize the social and historical determinants shaping Indigenous health.

It is important to clarify that key concepts such as coloniality and food coloniality were not operationalized as empirical variables within the quantitative analysis. Rather, these concepts function as theoretical-analytical lenses through which the observed statistical associations are interpreted. The study does not claim direct measurement of coloniality, nor does it infer causality between structural processes and individual-level outcomes. Instead, statistical findings are situated within broader historical and sociopolitical contexts affecting Indigenous territories and food systems.

It was conducted in 2022 with self-identified Indigenous adolescents aged 12–18 years, in accordance with the Brazilian Child and Adolescent Statute. Participants resided in villages of the Xukuru do Ororubá people, located in the Agreste mesoregion of the state of Pernambuco, northeastern Brazil (28).

### 2.2 Study sites and participant selection

Given the territorial extension of the Xukuru do Ororubá Indigenous Land and logistical considerations related to transportation and fieldwork, a purposive sampling strategy was adopted. From the list of 36 state-run Indigenous schools operating across the territory, one school from each of the three regions (Serra, Agreste, and Ribeira) was selected.

The inclusion criteria for schools were as follows: (i) official recognition as a state-run Indigenous school within the Xukuru education system; (ii) provision of education to adolescents aged 12–18 years; and (iii) the largest number of enrolled Indigenous adolescents within each respective region.

The inclusion criteria for adolescents were: (i) age between 12 and 18 years; (ii) self-identification as Indigenous Xukuru do Ororubá; (iii) enrollment in one of the selected schools; and (iv) residence within the Indigenous territory.

Based on these criteria, the following schools were included in the study: Ororubá School (Cana Brava village, Serra region); Monsenhor Olimpo Torres Intermediate School (Cimbres village, Agreste region); and Santa Rita School (Pé de Serra dos Nogueiras village, Ribeira region).

### 2.3 Sample size and sampling strategy

The estimated population of eligible adolescents was obtained from the Indigenous Health Care Information System (SIASI), which identified a total of 944 Indigenous adolescents aged 12–18 years residing in the Xukuru do Ororubá territory.

Sample size was calculated using the StatCalc module of Epi Info software, version 6.04 (Centers for Disease Control and Prevention, Atlanta, USA), applying the standard formula for finite populations. An expected prevalence of overweight of 26.3%, based on previous evidence among Brazilian adolescentes, was apoted (29). A 95% confidence level and a margin of error of ±5% were considered. Based on these parameters, the minimum required sample size was estimated at 226 adolescents.

In additional 10% was added to account for potential losses or refusals, resulting in an estimated sample of 249 participants.

Sampling was proportionally stratified by region according to the distribution of adolescents across the territory, resulting in the following allocation: 98 adolescents from the Serra region, 93 from the Agreste region, and 57 from the Ribeira region. Of the estimated sample, 225 adolescents completed all study procedures and were included in the final analysis.

### 2.4 Data collection and variables

Data were collected using structured questionnaires organized into thematic blocks covering demographic, socioeconomic, lifestyle, and anthropometric information.

Demographic and socioeconomic variables included Indigenous region of residence, sex (male/female), age, school shift, maternal schooling (years of education), number of household residents, number of rooms, housing characteristics, household income source, and drinking water treatment. Household economic status was assessed using the criteria of the Brazilian Association of Research Companies (ABEP), which classifies families into economic strata A, B1, B2, C1, C2, and D/E based on a point system. For analytical purposes, economic classes were grouped into two categories: ≥C2 and D/E (30).

Lifestyle variables included sedentary behavior, physical activity level, alcohol consumption, and smoking. Sedentary behavior was assessed based on daily screen time (use of electronic devices, computers, and television), with excessive screen time defined as more than two hours per day, according to the American Academy of Pediatrics (31).

Physical activity level was assessed using the International Physical Activity Questionnaire (IPAQ) (31). Physical activity performed in the week prior to the interview was evaluated based on weekly frequency and duration, and total accumulated minutes were calculated. The recommended level for adolescents is at least 60 min of moderate physical activity on five or more days per week, totaling a minimum of 300 min per week (32). For statistical analyses, participants were classified as physically active (≥300 min/week) or physically inactive (<300 min/week). Smoking was defined as current tobacco use, and alcohol consumption was categorized as “yes” or “no/rarely”.

Anthropometric measurements followed the technical standards of the Brazilian Food and Nutrition Surveillance System (SISVAN) (33). Body weight was measured using a digital scale (OMRON HBF-514C; capacity 150 kg; precision 100 g). Height was measured with a portable stadiometer (Altura Exata). All measurements were performed by a single trained evaluator to minimize inter-observer variability.

Two measurements of weight and height were obtained for each participant following standardized procedures. For height, if the difference between the two measurements exceeded 0.5 cm, a third measurement was taken, and the mean of the two closest values was used for analysis. When measurements were within the acceptable range, the mean of the two measurements was calculated. These procedures were adopted to ensure measurement accuracy and reliability (33).

Nutritional status was assessed using body mass index-for-age (BMI-for-age), calculated as weight (kg) divided by height squared (m^2^), and classified according to the WHO 2007 growth reference standards using Z-scores adjusted for sex and age. The following cut-off points were applied: Z-score ≥ − 3 and < −2 (thinness); ≥ − 2 and ≤ + 1 (normal weight); ≥ + 1 and < +2 (overweight); and ≥ + 2 and ≤ + 3 (obesity). For analytical purposes, adolescents with BMI-for-age Z-scores ≥ + 1 were classified as having overweight.

Participants were also asked about satisfaction with their current weight and their perception of weight status (very underweight/underweight, ideal, overweight/very overweight). For analysis, responses were grouped into three categories: very underweight/underweight, ideal, and overweight/very overweight.

All data collection procedures included verification of completeness and consistency of responses at the time of the interview. There were no missing data for key variables, as all inconsistencies and omissions were resolved during the data collection phase.

### 2.5 Statistical analysis

Anthropometric data were analyzed using AnthroPlus software, version 1.0.4 (World Health Organization, Geneva, Switzerland), and all statistical analyses were performed using IBM SPSS Statistics for Windows, version 13.0 (IBM Corp., Armonk, NY, USA). Associations between the outcome variable (overweight) and exposure variables (demographic, socioeconomic, and lifestyle factors) were initially assessed using Pearson’s chi-square test. Prevalence estimates were expressed as percentages, and statistical significance was set at *p* ≤ 0.05.

Prevalence ratios (PR) and their respective 95% confidence intervals (95% CI) were estimated using Poisson regression with robust variance. Variables presenting a *p*-value ≤ 0.25 in the bivariate analyses were selected for the multivariable analysis. The selection of variables for the multivariable models was guided by both statistical criteria (*p* ≤ 0.25 in bivariate analyses) and established theoretical frameworks on social determinants of adolescent overweight. Variables were organized hierarchically into distal (household and socioeconomic context), intermediate (maternal schooling), and proximal (individual behaviors and perceptions) levels, consistent with conceptual models commonly applied in epidemiological research. A hierarchical modeling strategy was adopted, with variables entered sequentially according to predefined conceptual levels: level 1 (number of household residents, household income source, and presence of a household toilet), level 2 (maternal schooling), and level 3 (age, school shift, alcohol consumption, and satisfaction with current weight).

All questionnaires were reviewed at the time of collection to minimize incomplete responses. As a result, no missing data were identified for the variables included in the analysis. Potential confounding was addressed through hierarchical multivariable modeling, with variables entered according to their conceptual level.

### 2.6 Ethical considerations and indigenous data governance

The study was approved by the Research Ethics Committee of the Federal University of Pernambuco (UFPE) and by the Brazilian National Research Ethics Commission (CONEP) (CAAE: 54457721.1.0000.5208). In addition to institutional approvals, the research respected the sociocultural protocols of the Xukuru do Ororubá people. Local leadership authorized the study, and all fieldwork was conducted with the direct accompaniment of a community member, ensuring continuous cultural mediation and community oversight.

In addition to formal ethical approval, this research was conducted in partnership with the Xukuru do Ororubá community and in accordance with Indigenous governance structures. Prior to data collection, the study objectives, procedures, and intended use of results were presented and discussed with Indigenous representatives from the education and health sectors within the territory. Authorization to conduct the research in Indigenous schools was granted following this dialogue.

Throughout the fieldwork, activities were accompanied by a designated community member, ensuring cultural mediation, transparency, and local oversight. The study was therefore conducted not merely with Indigenous individuals, but within an Indigenous community framework that recognizes collective authority and territorial governance.

Data use is restricted to the purposes described in this study. Any future use will require renewed consultation and authorization from the Xukuru community, in alignment with the CARE principles (Collective Benefit, Authority to Control, Responsibility, and Ethics), which guided the management and dissemination of findings.

### 2.7 Community feedback and dissemination

Study findings were returned to the territory through participatory and dialogical processes grounded in collective engagement. Rather than a unidirectional dissemination of results, the research team prioritized reciprocal knowledge exchange, ensuring that findings were shared, discussed, and interpreted within community spaces.

Results were presented at an extraordinary meeting of the Indigenous District Health Council (CONDISI), which brings together Indigenous leaders and community representatives, and were also shared with the Xukuru Local Health Council. These gatherings created opportunities for collective reflection on the implications of the findings for adolescent health, food practices, and territorial well-being.

By fostering shared interpretation and dialogue, these processes sought to ensure that the knowledge generated would return to the territory in a meaningful way, contributing to community-level reflection and decision-making, in alignment with Indigenous data governance principles.

### 2.8 Methodological reflexivity and epistemological positioning

Although internationally validated and widely used in public health research, instruments such as the IPAQ and WHO BMI-for-age criteria were developed primarily in non-Indigenous contexts. Their application in Indigenous territories may not fully capture culturally specific conceptions of body, health, physical activity, and well-being, nor the territorial and sociocultural dynamics shaping Indigenous adolescents’ lived experiences.

Their use in this study was pragmatic, given the absence of culturally validated epidemiological tools for Indigenous populations and the need for comparability with national and international health surveillance data that inform public policies affecting Indigenous peoples. The adoption of these instruments does not imply their universal cultural validity.

To address these tensions, quantitative findings were interpreted within a critical and dialogical framework informed by Xukuru knowledge systems and the principle of Bem Comer. By acknowledging these methodological constraints, the study seeks to contribute both to epidemiological evidence and to ongoing reflections on the development of culturally responsive research tools in Indigenous health contexts.

## 3 Results

A total of 9% of the initially eligible participants (*n* = 23) were lost due to school dropout or inconsistencies in questionnaire information. Thus, the final analytical sample comprised 225 Indigenous adolescents, including 87 from the Serra region, 83 from the Agreste region, and 55 from the Ribeira region. Of the total sample, 42.7% (*n* = 96) were male and 57.3% (*n* = 129) were female.

**Figure 1:**
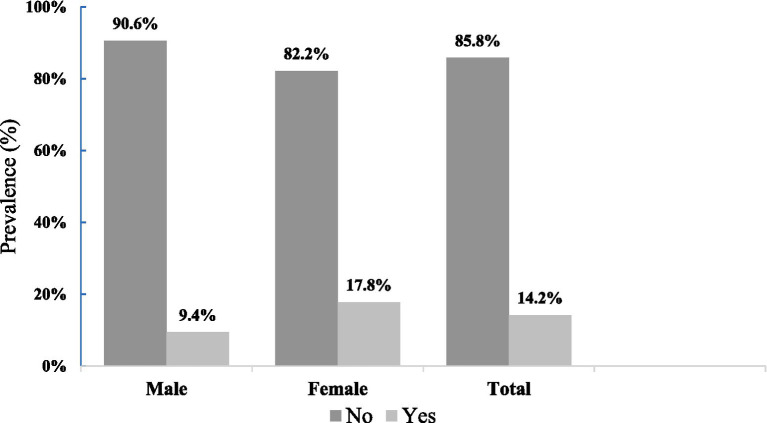
Prevalence of overweight among Xukuru do Ororubá adolescents by sex in Pernambuco state, Brazil, 2022.

As shown in Figure 1, the overall prevalence of overweight among adolescents was 14.2%, including 9.8% classified as overweight and 4.4% as obese. Overweight prevalence was higher among females (17.8%) compared with males (9.4%).

According to Table 1, 38.6% of the adolescents resided in the Serra region, 77.0% were aged 16–18 years, and 84.8% attended school during the afternoon shift. More than half of the participants (54.7%) were enrolled at Monsenhor Olimpo Torres Indigenous School, located in the Agreste region.

**Table 1:** Overweight among Xukuru do Ororubá adolescents according to demographic and socioeconomic factors in Pernambuco state, Brazil, 2022.

Variables	Total		Overweight				*p*-value
			Yes		No		
	*N* = 225	%	(*n* = 32)	%	(*n* = 193)	%	
Sex							
Male	96	42.7	9	9.4	87	90.6	
Female	129	57.3	23	17.8	106	82.2	0.73
Age (years)							
14–15	52	23.1	10	19.2	42	80.8	
16–18	173	76.9	22	12.7	151	87.3	0.23
Indigenous área of residence							
Serra	87	38.7	10	11.5	77	88.5	
Agreste	83	36.9	14	16.9	69	83.1	
Ribeira	55	24.4	8	14.5	47	85.5	0.60
School shift							
Afternoon	191	84.9	25	13.1	166	86.9	
Evening	34	15.1	7	20.6	27	79.4	0.24
Indigenous school							
Ororubá	59	26.2	9	15.3	50	84.7	
Monsenhor Olimpo Torres	123	54.7	17	13.8	106	86.2	
Santa Rita	43	19.1	6	14.0	37	86.0	0.96
Maternal education (years of schooling)							
≤ 8	117	52.0	11	9.4	106	90.6	
> 8	108	48.0	21	19.4	87	80.6	0.03
Number of household residents							
Up to 4	100	44.4	18	18.0	82	82.0	
5 or more	125	55.6	14	11.2	111	88.8	0.14
Household income source							
Paid work/retirement	74	32.9	8	10.8	66	89.2	
Sale of agricultural products/handicrafts	81	36.0	10	12.3	71	87.7	
Social programs	70	31.1	14	20.0	56	80.0	0.24
Economic classification							
≥ C2	67	29.8	7	10.4	60	89.6	
D e E	158	70.2	25	15.8	133	84.2	0.29
Toilet facilities							
Inside the household	177	78.7	21	11.9	156	88.1	
Outside the household/shared	48	21.3	11	22.9	37	77.1	0.05
Water supply							
Public water supply	89	39.6	13	14.6	76	85.4	
Well or spring	136	60.4	19	14.0	117	86.0	0.90
Street surface							
Paved	20	8.9	2	10.0	18	90.0	
Dirt/gravel	205	91.1	30	14.6	175	85.4	0.57

Regarding household socioeconomic characteristics, 70.2% of adolescents belonged to economic classes D or E, and the predominant household income source was the sale of agricultural or artisanal products (36.0%). Maternal schooling of eight years or less was reported for 52.0% of participants. More than half of the adolescents (55.6%) lived in households with five or more residents. In bivariate analyses, no statistically significant associations were observed between these socioeconomic variables and overweight, except for maternal schooling. Adolescents whose mothers had more than eight years of education showed a higher prevalence of overweight (*p* = 0.03) (Table 1).

With respect to housing conditions, 21.3% of adolescents lived in households with a toilet located outside the home or shared with other households. Only 8.9% reported living on paved or asphalted streets, and 60.4% relied on wells or natural springs as their primary source of drinking water. No statistically significant associations were identified between these housing characteristics and overweight in the bivariate analysis (Table 1).

Lifestyle characteristics are presented in Table 2. Overall, 61.3% of adolescents were classified as sedentary, and only 10.8% reported engaging in 300 min or more of physical activity per week. Alcohol consumption and smoking were reported by 17.3 and 1.3% of participants, respectively. Excessive screen time (>2 h/day) was reported by 72.0% of adolescents. Variables significantly associated with overweight were dissatisfaction with current weight and perceived weight status, both showing strong statistical significance (*p* < 0.001). Adolescents who reported dissatisfaction with their weight and perceived themselves as overweight or very overweight presented a higher prevalence of overweight.

**Table 2:** Overweight among Xukuru do Ororubá adolescents according to lifestyle factors in Pernambuco state, Brazil, 2022.

Variables	Total		Overweight				*p*-value
			Yes		No		
	*N* = 225	%	(*n* = 32)	%	(*n* = 193)	%	
Physical activity (minutes/week)							
≥ 300 min	23	10.2	3	13.0	20	87.0	
< 300 min	202	89.8	29	14.4	173	85.6	0.86
Physical activity level (IPAQ)							
Sedentary/irregularly active	138	61.3	20	14.5	118	85.5	
Active/very active	87	38.7	12	13.8	75	86.2	0.88
Alcohol consumption							
Yes	39	17.3	3	7.7	36	92.3	
No/rarely	186	82.7	29	15.6	157	84.4	0.15
Smoking							
Yes	3	1.3	0	0.0	3	100.0	
No	222	98.7	32	14.4	190	85.6	0.47
Screen time exposure							
> 2 h/day	162	72.0	24	14.8	138	85.2	
≤ 2 h/day	63	28.0	8	12.7	55	87.3	0.68
Satisfaction with current weight							
Yes	163	72.4	13	8.0	150	92.0	
No	62	27.6	19	30.6	43	69.4	<0.001
Perception of body weight							
Very underweight/underweight	36	16.0	2	5.6	34	94.4	
Ideal	149	66.2	8	5.4	141	94.6	
Overweight/very overweight	40	17.8	22	55.0	18	45.0	<0.001

In the multivariable analysis (Table 3), the factors that remained significantly associated with overweight after adjustment were the presence of a household toilet (prevalence ratio [PR] = 2.4; 95% CI: 1.05–5.87; *p* = 0.03), maternal schooling greater than eight years (PR = 2.7; 95% CI: 1.21–6.37; *p* = 0.01), attendance in the night school shift (PR = 3.2; 95% CI: 1.07–9.90; p = 0.03), and dissatisfaction with current weight (PR = 6.6; 95% CI: 2.74–15.88; *p* < 0.001). Among these variables, dissatisfaction with current weight showed the strongest association with overweight, indicating that adolescents who were dissatisfied with their weight had approximately six times higher prevalence of overweight compared with those who reported being satisfied.

**Table 3:** Crude and adjusted prevalence ratios for overweight according to socioeconomic, demographic, and lifestyle variables among Xukuru do Ororubá adolescents in Pernambuco state, Brazil, 2022.

Variables	Total			Overweight				*p*-value
				Crude PR		Adjusted PR		
	*N* = 225	*N* = 32	%	PR	95% CI	PR	95% CI	
Level 1∆								
Number of household residents								
5 or more	125	14	11.2	1.0		1.0		
Up to 4	100	18	18.0	1.7	0.81–3.70	2.0	0.94–4.62	0.06
Household income source								
Paid work/retirement	74	8	10.8	1.0		1.0		
Sale of agricultural products/handicrafts	81	10	12.3	1.1	0.43–3.12	1.1	0.42–3.18	
Social programs	70	14	20.0	2.0	0.80–5.27	1.9	0.74–5.07	0.17
Toilet facilities								
Inside the household	177	21	11.9	1.0		1.0		
Outside the household	48	11	22.9	2.2	0.98–4.90	2.4	1.05–5.87	**0.03**
Level 2◊								
Maternal education								
≤ 8 years	117	11	9.4	1.0		1.0		
> 8 years	108	21	19.4	2.3	1.06–5.08	2.7	1.21–6.37	**0.01**
Level 3□								
Age (years)								
16–18	173	22	12.7	1.0		1.0		
14–15	52	10	19.2	1.6	0.71–3.71	2.2	0.85–6.17	0.09
School shift								
Afternoon	191	25	13.1	1.0		1.0		
Evening	34	7	20.6	1.7	0.67–4.37	3.2	1.07–9.90	**0.03**
Alcohol consumption								
No/rarely	186	29	15.6	1.0		1.0		
Yes	39	3	7.7	2.2	0.64–7.68	2.1	0.54–8.66	0.27
Satisfaction with current weight								
Yes	163	13	8.0	1.0		1.0		
No	62	19	30.6	5.0	2.33–11.15	6.6	2.74–15.88	**<0.001**

∆ Level 1: socioeconomic variables; ◊ Level 2: adjusted for Level 1 variables; □Level 3: adjusted for Level 1 and Level 2 variables. Note: Bold values indicate statistically significant results (*p* < 0.05).

## 4 Discussion

This study identified a prevalence of overweight of 14.2% among adolescents of the Xukuru do Ororubá people in Pernambuco, Brazil. Although the prevalence of overweight varies across Indigenous peoples and even among communities within the same ethnic group, findings lower than those observed in the non-Indigenous Brazilian population are not uncommon (34–37). These differences, however, should not be interpreted solely through biological or behavioral perspectives.

The variations observed reflect, above all, differing degrees of territorial preservation, food autonomy, and continuity of traditional systems of care. Understanding these findings requires situating Indigenous adolescents’ nutritional status within the historical processes that have shaped Indigenous food systems and ways of living (38, 39).

Historically, Indigenous food practices in Brazil were grounded in autonomous, sustainable systems closely integrated with territory, based on hunting, fishing, and traditional agriculture. Colonial processes introduced ultra-processed foods, monoculture practices, and the devaluation of traditional food knowledge, generating profound ruptures in relationships of reciprocity with nature. These processes express food coloniality, which continues to shape the nutritional status of contemporary Indigenous populations and helps explain the emergence of overweight in diverse Indigenous contexts (39).

Although similar patterns of overweight have been described among non-Indigenous adolescents in Latin America, the United States, and municipalities in Pernambuco State, such comparisons must be made with caution (40–42). Among Indigenous peoples, overweight cannot be dissociated from processes of deterritorialization, the weakening of traditional food systems, and the forced reorganization of social time and daily life.

The findings of this study are consistent with those reported by Rodrigues (43), who identified the coexistence of traditional practices and dietary patterns characteristic of the nutritional transition among Xukuru adults. However, the scarcity of studies focusing on Indigenous adolescents highlights a significant gap in the literature and reinforces the need to consider adolescence as a critical life stage marked by tensions between ancestral knowledge and contemporary external influences (44).

Continuous contact with non-Indigenous society has weakened traditional practices of care, food production, and social organization, contributing to the reconfiguration of Indigenous epidemiological profiles and the emergence of chronic conditions such as obesity, hypertension, and diabetes. This scenario is further aggravated by territorial invasions and illegal exploitation of natural resources, which generate environmental degradation, conflict, and long-term restrictions on land use (44). Thus, observed nutritional outcomes must be interpreted in light of broader structural determinants.

In this study, unfavorable social and economic conditions were evident, including precarious housing, inadequate sanitation, and limited access to safe drinking water. The association between socioeconomic vulnerability and the rise of overweight has been documented in previous studies, reinforcing the coexistence of food insecurity and nutritional disorders in vulnerable populations (45, 46).

Among the factors associated with overweight, household sanitation remained significant in the multivariable model. Adolescents living in households with toilets located outside the home or shared collectively showed a higher prevalence of overweight. Many of these families are beneficiaries of the Bolsa Família Program, for which the literature reports higher levels of food insecurity and implications for regular and adequate access to food (47–49).

Beyond economic constraints, the loss of food sovereignty emerges as a central structural determinant of nutritional insecurity among Indigenous peoples. Limited access to land and traditional food production compromises the right to *Bem Comer*, a concept that among the Xukuru people transcends nutritional adequacy and encompasses spiritual, cultural, and collective dimensions of food. The erosion of this right affects not only nutritional status but also fundamental relationships between body, territory, and community.

Maternal schooling greater than eight years was also associated with a higher prevalence of overweight among adolescents. Although parental education is generally associated with better health outcomes, studies conducted in contexts of nutritional transition suggest that this relationship may be reversed (50–52). In populations experiencing urbanization and rapid dietary change, higher schooling may coexist with increased exposure to Westernized lifestyles and ultra-processed foods.

School shift was another factor significantly associated with overweight, with higher prevalence observed among adolescents attending night school. Although many of these students engage in occupational activities during the day, no direct association was identified between physical activity level and overweight. This finding suggests that overweight is less related to isolated individual behaviors and more to disruptions in communal cycles of eating, work, rest, and social interaction, resulting from overlapping school and labor demands that conflict with traditional Indigenous temporal organization.

Body perception emerged as the variable most strongly associated with overweight. Adolescents dissatisfied with their current weight presented a significantly higher prevalence of the outcome. Among Indigenous peoples, the body is intrinsically connected to ways of living and ancestry, integrating mind, body, and spirit. Body dissatisfaction, widely documented in Western societies, reflects cultural and social pressures that also permeate Indigenous territories, imposing aesthetic standards that devalue collective and Indigenous corporealities (53–56).

Although not statistically associated with overweight, a high prevalence of excessive screen time was observed. This phenomenon may be related to the increasing presence of the internet and social media, which, while altering traditional dynamics, have also been appropriated by Indigenous youth as spaces for cultural affirmation (57).

Understanding changes in dietary patterns, body perceptions, and lifestyles among Xukuru do Ororubá adolescents requires moving beyond strictly biomedical interpretations and recognizing the persistent effects of coloniality across multiple dimensions of Indigenous existence. As argued by Santos and Meneses (2010), coloniality continues to operate through “abyssal lines” that deny the legitimacy of diverse ways of knowing and being, constituting a central element of colonial power (58).

In this context, Xukuru do Ororubá ways of living emerge as structuring pathways of care and healing. Their cosmological conceptions of health, transmitted intergenerationally, articulate territory, collectivity, food, and the balance between mind, body, and spirit in harmony with nature (59, 60). Practices such as annual reforestation of native seedlings, the exchange of traditional seeds, collective commensality, and *Bem Comer*, express Indigenous systems of care that strengthen identity and promote well-being.

For the Xukuru people, food is regarded as a sacred element and is celebrated daily through the practice of Bem Comer, a concept rooted in resistance, historical continuity, and territorial sustainability. Bem Comer expresses an Indigenous principle of health that integrates food, spirituality, land, and collective life.

Within the scope of this study, Bem Comer is not treated as a measurable practice or as a specific intervention. Rather, it is understood primarily as a normative and epistemological Indigenous principle of care that informs how health and well-being are conceived within the Xukuru territory. Methodologically, it functions as an analytical lens guiding the critical interpretation of the epidemiological findings, particularly in relation to food coloniality and the structural determinants of Indigenous health.

Addressing overweight among Xukuru adolescents cannot be dissociated from these healing practices, which also operate as forms of resistance to ongoing coloniality. By strengthening Indigenous ways of being, caring, and producing health, these practices contribute to identity reconstruction among younger generations.

Finally, the findings highlight the need for public policies that integrate food, culture, and territory, recognizing Indigenous peoples’ right to food self-determination. Culturally grounded Food and Nutrition Education programs, aligned with traditional knowledge and implemented in school settings, may contribute to reducing nutritional disorders and strengthening local practices of care and healing.

Given the cross-sectional design, causal relationships cannot be inferred, and reverse causality cannot be ruled out. Additional limitations include the use of the IPAQ in an Indigenous community, which, although validated for the Brazilian population, may present constraints in this specific cultural context, and the conduction of the study in a single Indigenous territory, limiting generalizability. These limitations reinforce the importance of territorialized, culturally situated analyses conducted in dialogue with Indigenous peoples.

The findings of this study are contextually grounded in the Xukuru do Ororubá territory and should not be interpreted as statistically generalizable to other Indigenous peoples in Brazil or elsewhere. Indigenous populations are characterized by profound historical, territorial, sociopolitical, and cosmological diversity, which shapes distinct health dynamics across contexts.

Rather than offering universal claims about Indigenous adolescents, this study provides a territorially situated epidemiological analysis, interpreted within the specific structural and cultural realities of the Xukuru people. Its contribution lies in illustrating how quantitative findings can be meaningfully contextualized within Indigenous knowledge systems, rather than in establishing generalizable patterns across diverse Indigenous populations.

## 5 Conclusion

This study showed that overweight represents a relevant health concern among Xukuru do Ororubá adolescents, with a prevalence of 14.2%, and that its occurrence is shaped by socioeconomic conditions, household infrastructure, school time organization, and body perception. These findings demonstrate that understanding overweight among Indigenous adolescents cannot be limited to biomedical or behavioral approaches alone within this specific territorial and sociocultural context.

By articulating epidemiological data with an analysis grounded in Indigenous knowledge, the study contributes to interpreting overweight as an expression of food coloniality, linked to structural transformations in territory, food systems, and Indigenous ways of living. These interpretations are analytically situated in the Xukuru territory and do not imply universal generalizations across Indigenous populations.

From a public health standpoint, the results highlight the need for policies and actions that integrate food, territory, and culture, recognizing Indigenous peoples’ right to food self-determination. Food and Nutrition Education strategies developed in dialogue with Indigenous knowledge and implemented in school and community settings are essential for addressing nutritional challenges during Indigenous adolescence.

Finally, this study demonstrates that epidemiological research, when conducted through ethical and dialogical approaches, can move beyond the description of health outcomes to support local practices of care and health production. By integrating quantitative analysis with Indigenous epistemologies, the study offers valuable insights for adolescent health approaches aligned with cultural diversity, social justice, and the principles of *Indigenous Approaches for Healing: Wisdom and Best Practices from All Our Relations*.

## Data Availability

The datasets presented in this study can be found in online repositories. The names of the repository/repositories and accession number(s) can be found: https://repositorio.ufpe.br/handle/123456789/51234.
